# Does robotics add value in Hartmann’s reversal? A PRISMA-based systematic review and meta-analysis of perioperative outcomes

**DOI:** 10.1007/s10151-026-03414-5

**Published:** 2026-08-14

**Authors:** A. Abdelsamad, Y. Khalil, K. Mohamed, A. Idriss Ibrahim, A. Elgamasy, K. A. Abdelrahman, T. Sprenger, T. A. A. M. Habeeb, T. Herzog, F. Gebauer

**Affiliations:** 1 Department of Surgery II, University of Witten/Herdecke, Witten, Germany; 2 Knappschaft Klinikum-Vest, Dorstenerstr. 151, 45657 Recklinghausen, Germany; 3 Faculty of Medicine, Capital University, Cairo, Egypt; 4 Department of Surgery, The Ohio State University Wexner Medical Center and James Comprehensive Cancer Center, Columbus, OH USA; 5 Medical intern, Faculty of Medicine, Cairo University, Cairo, Egypt; 6 Faculty of Medicine, Cairo University, Cairo, Egypt; 7 Department of Bariatric Surgery, Knappschaft-Hospital, Recklinghausen, Germany; 8 Department of General Surgery, Faculty of Medicine, Zagazig University, Zagazig, Egypt; 9 Department of Surgery, Ruhr University of Bochum, Bochum, Germany; 10 Department of Surgery, Helios University Hospital Wuppertal, Wuppertal, Germany

**Keywords:** Hartmann reversal, Laparoscopic, Robotic-assisted, Perioperative outcomes, Meta-analysis

## Abstract

**Background:**

Reversal of Hartmann’s procedure is technically demanding and occasionally performed via open surgery, which is associated with substantial morbidity. Minimally invasive approaches have expanded, yet comparative evidence between robotic and laparoscopic reversal remains limited. We conducted a systematic review and meta-analysis to compare perioperative outcomes of both approaches.

**Methods:**

Databases were searched through 20 July 2026. Studies reporting outcomes after laparoscopic and/or robotic Hartmann’s reversal were included. Random-effects meta-analyses were performed with preplanned subgroup comparisons (robotic versus laparoscopic). Pooled estimates were reported with 95% confidence intervals; heterogeneity was assessed using *I*^2^. Evidence was assessed using Grading of Recommendations, Assessment, Development and Evaluation (GRADE).

**Results:**

Sixty-five studies comprising >10,000 patients were included. Operative time was significantly longer with robotics (274.6 versus 176.04 min; *P* = 0.018). Robotic studies reported lower pooled surgical site infection rates (4% versus 6%; *P* = 0.02) and shorter length of hospital stay (4.2 versus 8.0 days; *P* < 0.001). Conversion to open surgery pooled rate was lower in robotic studies (7% versus 14%), yet the subgroup difference did not reach statistical significance.

**Conclusions:**

Robotic Hartmann’s reversal was associated with longer operative time, while pooled analyses showed lower surgical site infection rates and shorter hospital stay than pooled laparoscopic reversal. However, the current robotic evidence base remains limited and is largely derived from indirect comparisons and pooled single-arm analyses. Higher-quality comparative data are needed to define patient selection, cost, and benefit.

**Supplementary Information:**

The online version contains supplementary material available at 10.1007/s10151-026-03414-5.

## Introduction

Hartmann’s procedure remains a common operation in colorectal surgery, typically performed for complicated diverticulitis, obstructing or perforated colorectal cancer, ischemia, or severe pelvic sepsis where primary anastomosis is considered unsafe [1]. While the index operation can be life-saving, the creation of an end colostomy carries a significant long-term burden for patients, including stoma-related complications (including prolapse, bleeding, retraction, abscess, etc.), body-image concerns, reduced quality of life, and limitations in daily activities. Consequently, reversal of Hartmann’s procedure, restoring intestinal continuity, represents a clinically important step toward functional recovery in appropriately selected patients [2]. However, Hartmann’s reversal is widely recognized as one of the most demanding restorative colorectal procedures due to adhesions, altered anatomy, and the technical complexity of pelvic dissection and colorectal anastomosis [3].

Traditionally, Hartmann’s reversal has been performed via an open approach. While minimal invasive surgery (MIS) is now the preferred first-line strategy, open reversal is still undertaken and may remain the only suitable option for selected patients in complex or high-risk scenarios [4]. However, open reversal is associated with considerable morbidity, particularly wound-related complications such as surgical site infection, fascial dehiscence, and subsequent incisional hernia formation, which can prolong recovery, increase reinterventions, and escalate healthcare utilization [5].

These risks are amplified in a population that often presents with prior sepsis, multiple comorbidities, and a hostile abdomen from previous laparotomy. In this context, open surgery has increasingly become an “ultima ratio” option, reserved for highly complex cases or when minimally invasive strategies are not technically achievable or safe [6].

Over the last two decades, (MIS) has increasingly been adopted for Hartmann reversal, even when the index Hartmann operation was performed open [7]. Laparoscopic reversal offers potential advantages through reduced abdominal wall trauma, improved visualization, and a lower wound burden, translating into fewer wound complications and shorter convalescence in many elective colorectal settings [8]. Nonetheless, laparoscopy in Hartmann reversal remains technically challenging: dense adhesiolysis, splenic flexure mobilization, and pelvic dissection can be demanding, and conversion to open surgery is not uncommon, particularly in patients with extensive adhesions or difficult pelvic anatomy. Therefore, the extent to which laparoscopy consistently delivers “full MIS” benefits in this setting remains a key question [3, 9].

The integration of robotic platforms has further expanded the MIS options. Robotic surgery provides stable high-definition three-dimensional (3D) visualization, wristed instrumentation, tremor filtration, and improved ergonomics, which may facilitate meticulous adhesiolysis, intracorporeal suturing, and dissection in confined spaces [10]. In colorectal surgery, robotic approaches have been associated in several contexts with lower conversion rates, reduced surgical site infection, and shorter length of stay, albeit often at the expense of longer operative time and higher direct costs [11]. These trade-offs are particularly relevant in Hartmann reversal, where resource intensity is high and institutional volume, team experience, and patient selection may substantially influence outcomes and value [6, 12].

Despite increasing adoption, the comparative evidence base for robotic versus laparoscopic Hartmann reversal remains limited. To address this gap, we performed a systematic review and meta-analysis to synthesize the available data and compare perioperative outcomes of robotic-assisted versus laparoscopic Hartmann reversal. We aimed to provide an evidence-based framework to guide approach selection, inform patient counseling, and clarify the potential incremental value of robotics in this demanding restorative procedure.

## Materials and methods

### Ethical approval

This systematic review and meta-analysis was conducted in accordance with the methodological standards outlined by the Cochrane Collaboration and followed the guidelines stated by the Preferred Reporting Items for Systematic Reviews and Meta-Analyses (PRISMA) [13, 14]. In addition, the manuscript followed AMSTAR 2 guidelines to evaluate the methodological quality of the present review [15]. This review was registered in prospective register of ongoing systematic protocols of evaluation and recommendations (PROSPERO), with the reference number of (CRD420261324990) [16]. PRISMA checklist is available in Supplementary files Table 1S.

### Search strategy

**Fig. 1:**
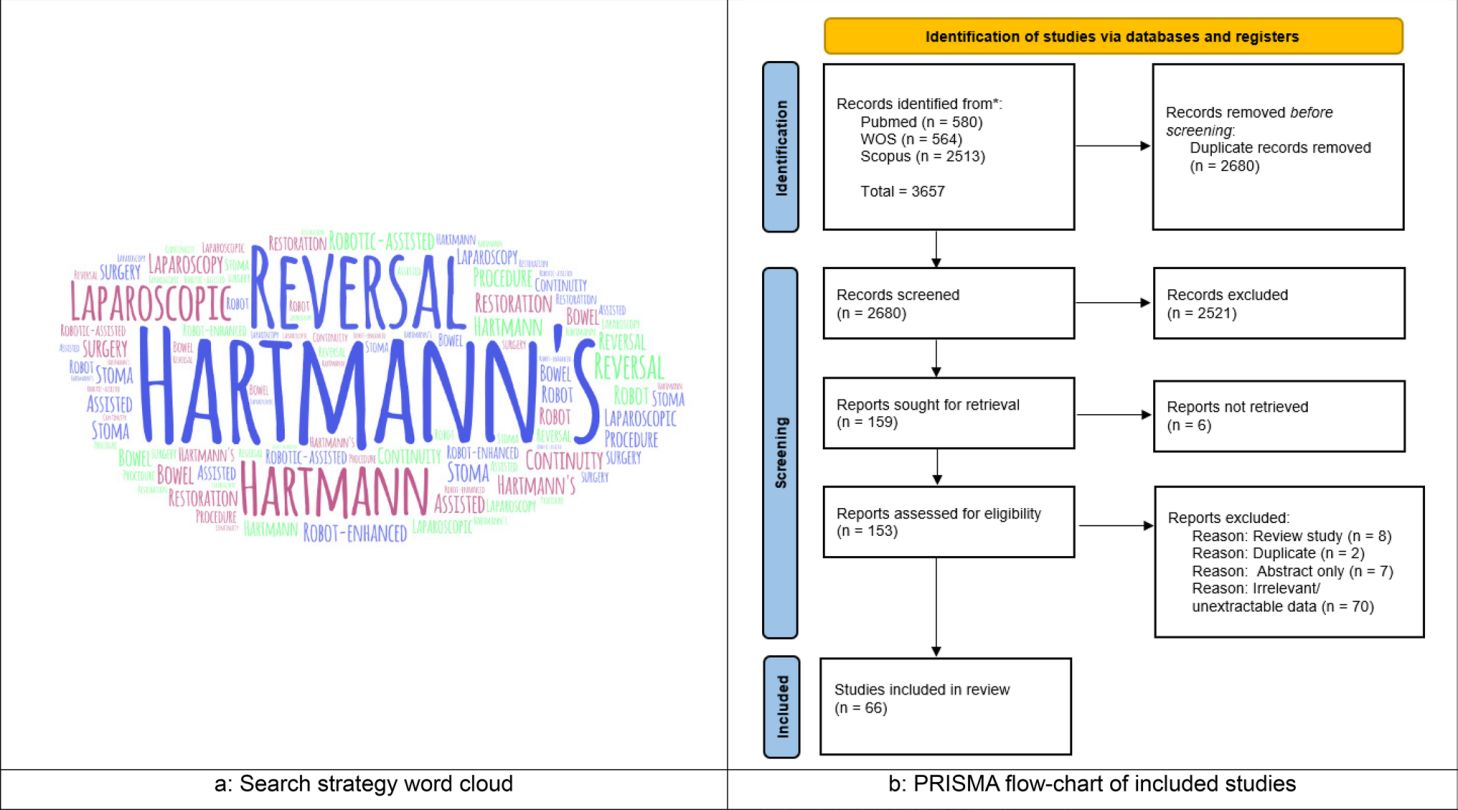
Search strategy

Medical Subject Headings (MeSH) terms and other relevant medical terms from relevant studies were used to formulate a robust search strategy using terms such as “Hartmann reversal,” “Laparoscopy,” and “Robotic-Assisted Surgery.” Literature search was conducted across three major electronic databases, including PubMed, Scopus, and Web of Science, to identify all relevant studies published up to 20 July 2026. To ensure comprehensive coverage and optimize sensitivity, the search strategy employed an extensive range of relevant keywords and synonymous terms related to Hartmann’s reversal surgery (including “stoma reversal” OR “Hartmann reversal” OR “reversal of Hartmann” OR “restoration of bowel continuity” AND “laparoscopy” OR “laparoscopic Assisted Surgery” OR “laparoscopic surgery” OR “robotic-assisted surgery” OR “robot surgery” OR “robot-enhanced procedure”), as per Fig. 1A—word cloud.

### Study design and outcomes (PICOS Framework)

This review followed the guidelines listed by the framework of Population, Intervention, Comparator, Outcomes, Study design (PICOS) criteria [17]. The population included patients undergoing surgery for Hartmann’s reversal. Intervention is patients undergoing Hartmann surgery reversal by a laparoscopic approach; however, the comparator is patients undergoing Hartmann surgery reversal by robotic assisted approach. The outcomes sought to be evaluated included overall morbidity, postoperative complications, and length of hospital stay.

### Study selection and screening

The inclusion criteria included primary studies with PICOS as mentioned (cross-sectional, case-control, cohort, case series, and randomized controlled trial with available data relevant to our PICO criteria). However, the exclusion criteria were: (1) case reports, review articles, and abstracts; (2) reviews, meta, commentary, editorials, letters, or conference abstracts without full-text availability; (3) in vitro or animal studies or studies conducted on nonhuman subjects; (4) laboratory studies; and (5) studies with inadequate information on outcomes or where relevant outcome measures are missing. The collected database search was imported into Zotero to remove duplicates [18]. Afterward, four reviewers screened the database search independently by title and abstract and by full text. Discrepancies or disagreements were resolved by a fifth senior reviewer. Fig. 1B shows the PRISMA flowchart.

### Data extraction and outcomes

Two reviewers worked independently to extract data about the different outcomes sought to be evaluated from the included studies. Simultaneously, two reviewers worked independently to extract data about the studies and patients’ characteristics. Any discrepancies or disagreements were resolved by a senior reviewer.

### Quality assessment

The quality of the included studies was evaluated using two tools. Case series studies were tested using the NHLBI quality assessment tool for case series studies [19]. It consists of nine different questions to be answered for each study with a yes, no, cannot determine, not applicable, or not reported answers; it is then to be rated as good, fair, or poor-quality rating according to the reviewer’s judgment. On the other hand, Newcastle–Ottawa quality assessment scale (NOS) was used for cohort studies [20]. This instrument assesses the risk of bias and internal validity of observational studies across three domains (selection, comparability, and outcome assessment) with maximum scores of four, two, and three stars, respectively, as presented in Table 1.

**Table 1:** Quality assessment of included studies

A: Newcastle-Ottawa Scale cohort studies									
	Selection				Comparabilty	Outcome			Total
	Representativeness of exposed cohort	Selection of nonexposed cohort	Ascertainment of exposure	Outcome not present at the start of the study		Assessment of outcomes	Length of follow-up	Adequacy of follow-up	
Abueta 2022	★	★	★	★	★★	★	★	★	9
Achkasov 2010	★	★	★	★	★	★	★	★	8
Akmercan 2025	★	★	★	★	★	★	★	★	8
Aquina 2016	★	★	★	★	★★	★	★	★	9
Arkenbosch 2015	★	★	★	★	★★	★	★	★	9
Brathwaite 2015	★	★	★	★	★	★	★	★	8
Cassini 2017	★	★	★	★	★★	★	★	★	9
Cellini 2013	★	★	★	★	★★	★	★	★	9
Chen 2021	★	★	★	★	★★	★	★	★	9
Cho 2021	★	★	★	★	★	★	★	★	8
Clermonts 2016	★	★	★	★	★	★	★	★	8
De’Angelis 2013	★	★	★	★	★★	★	★	★	9
Faure 2007	★	★	★	★	★★	★	★	★	9
Goncharov 2023	★	★	★	★	★	★	★	★	8
Haughn 2008	★	★	★	★	★★	★	★	★	9
Horesh 2018	★	★	★	★	★★	★	★	★	9
Katsura 2024	★	★	★	★	★★	★	★	★	9
Kwak 2018	★	★	★	★	★★	★	★	★	9
Lee 2023	★	★	★	★	★★	★	★	★	9
Mazeh 2009	★	★	★	★	★★	★	★	★	9
Melkonian 2017	★	★	★	★	★	★	★	★	8
Minatti 2022	★	★	★	★		★	★	★	7
Mirza 2021	★	★	★	★	★	★	★	★	8
Misra 2022	★		★	★		★	★	★	6
Ng 2013	★	★	★	★	★	★	★	★	8
Nguyen 2022	★		★	★		★	★	★	6
Onder 2016	★	★	★	★	★★	★	★	★	9
Park 2018	★	★	★	★	★	★	★	★	8
Pei 2018	★	★	★	★	★★	★	★	★	9
Petersen 2009	★		★	★		★	★	★	6
Reali 2022	★	★	★	★	★	★	★	★	8
Rosen 2005	★		★	★		★	★	★	6
Sato 2022	★	★	★	★	★	★	★	★	8
Steinemann 2015	★	★	★	★	★★	★	★	★	9
Tabibian 2025	★	★	★	★	★★	★	★	★	9
Walklett 2014	★	★	★	★	★	★	★	★	8
Whitney 2020	★	★	★	★	★	★	★	★	8
Yang 2014	★	★	★	★	★★	★	★	★	9
Zimmermann 2014	★	★	★	★	★	★	★	★	8
Imigo 2022	★	★	★	★	★★	★	★	★	9
Brathwaite 2017	★	★	★	★	★★		★	★	8
Ottaviano 2023	★	★	★	★	★	★	★	★	8
Ferrari 2024	★	★	★	★	★	★	★	★	8
Tan 2023	★	★	★	★	★★	★	★	★	9
Manigrasso 2026	★	★	★	★	★★	★	★	★	9

B: Newcastle-Ottawa Scale case-control studies									
	Selection				Comparability	outcome			Total
	Case definition adequate	Representativeness of cases	Selection of control	Definition of controls		Assessment of exposure	Same method of ascertainment for cases and controls	Nonresponse rate	
D’Alessandro 2020	★	★	★	★	★★	★	★	★	9
Maitra 2013	★	★	★	★	★	★	★		7

C: NIH quality assessment tool for case series studies										
Study	Q1	Q2	Q3	Q4	Q5	Q6	Q7	Q8	Q9	Quality rating
Akmercan 2024	Yes	Yes	Yes	Yes	Yes	Yes	Yes	Yes	Yes	Good
Bottger 2011	Yes	Yes	Yes	Yes	Yes	Yes	Yes	Yes	Yes	Good
Carus 2008	Yes	Yes	Yes	Yes	Yes	Yes	Yes	No	Yes	Fair
Caselli 2010	Yes	Yes	Yes	Yes	Yes	Yes	Yes	N/A	Yes	Fair
Choi 2015	Yes	Yes	Yes	Yes	Yes	Yes	Yes	No	Yes	Good
Chouillard 2007	Yes	Yes	Yes	Yes	Yes	Yes	Yes	No	Yes	Fair
Froehlich 2025	Yes	Yes	Yes	Yes	Yes	Yes	Yes	Yes	Yes	Good
Giuliani 2020	Yes	Yes	Yes	Yes	Yes	Yes	Yes	Yes	Yes	Good
Giuseppe 2019	Yes	Yes	Yes	Yes	Yes	Yes	Yes	N/A	Yes	Good
Golash 2006	Yes	Yes	Yes	Yes	Yes	Yes	Yes	N/A	Yes	Good
Kang 2021	Yes	Yes	Yes	Yes	Yes	Yes	CD	Yes	Yes	Fair
Leroy 2011	Yes	Yes	Yes	Yes	Yes	Yes	CD	Yes	Yes	Fair
Fiscon 2014	Yes	Yes	Yes	Yes	Yes	Yes	Yes	N/A	Yes	Good
Thambi 2021	Yes	Yes	Yes	Yes	Yes	Yes	no	Yes	Yes	Good
Svenningsen 2010	Yes	Yes	Yes	Yes	Yes	Yes	yes	Yes	Yes	Good
Vacher 2002	Yes	Yes	Yes	Yes	Yes	Yes	yes	Yes	Yes	Good
Van Loon 2020	Yes	Yes	Yes	Yes	Yes	Yes	Yes	Yes	Yes	Good

### Certainty of evidence

The certainty of each outcome in this review was graded using the Grading of Recommendations, Assessment, Development and Evaluation (GRADE) framework [21]. The framework evaluates certainty on the basis of five key domains (risk of bias, indirectness, inconsistency, imprecision, and publication bias) and subsequently classifies the confidence in our proposed outcomes as high, moderate, low, or very low.

### Statistical analysis

A comprehensive meta-analysis was conducted to synthesize continuous and dichotomous outcomes comparing Hartmann’s reversal using laparoscopic and robotic-assisted procedures. All statistical analyses were performed using RStudio using meta and metafor packages [22, 23]. Random effect was applied to account for expected heterogeneity among included studies [24].

Pre-planned subgroup analyses were performed to compare outcomes between laparoscopic and robotic surgical approaches. For outcomes that had two or more studies with direct comparison between both approaches, a formal direct comparative meta-analysis was done. However, for outcomes with insufficient comparative data, pooled single-arm meta-analysis were performed for each approach with preplanned subgroup comparison.

Subgroup differences were tested using the Chi-squared test for subgroup differences, an approach proposed by Richardson et al. 2019, which evaluates whether the effect sizes differ significantly between subgroups [25]. The subgroup analyses were conducted within the same random-effects framework. All pooled estimates are reported with 95% confidence intervals. Statistical significance for subgroup differences was assessed at *P* < 0.05. Leave-one-out sensitivity analysis and funnel plots with Egger’s test were generated to test for heterogeneity and publication bias [26].

Heterogeneity was quantified using the *I*^2^ statistic, which describes the percentage of total variation across studies attributable to heterogeneity rather than chance. For outcomes in which only a single study contributed to a study arm, *I*^2^ was not estimable and is therefore reported as not applicable (N/A).

## Results

By searching PubMed, Web of Science, and Scopus, 3657 search results in total were collected; 2680 duplicates were removed. Overall, 153 records were screened for eligibility for inclusion in our analysis (Fig. 1B). A total of 65 studies [3, 6, 7, 12, 27–34, 34, 34–86] were included in this review. The included studies comprised more than 10,000 patients who underwent reversal of Hartmann’s procedure, including 60 studies evaluating laparoscopic reversal and 5 studies evaluating robotic reversal. Among the robotic studies, three provided direct comparative analyses between robotic and laparoscopic approaches, whereas the remaining two reported single-arm outcomes for robotic reversal only.

Outcomes were stratified by the type of approach used for the procedure, either laparoscopic or robotic. Among the included robotic studies, three [6, 7, 86] provided direct comparative data with laparoscopic approach, allowing direct meta-analysis for operative time, conversion to open approach, postoperative morbidity, and anastomotic leak outcomes. The remaining outcomes were evaluated using pooled single-arm analysis with subgroup comparison. Random effect was applied to all analyses to account for the substantial heterogeneity observed across studies.

### Baseline Characteristics of the included studies

The studies included in the present meta-analysis comprised predominantly retrospective cohort studies (44 studies), along with 19 case series and 3 prospective cohort studies; except for one study, most of the studies were nonfunded (Table 2). The mean age of the included patients ranged from 40 to 75 years, and the percentage of male patients ranged from 30% to 66%. Other information regarding patients’ comorbidities, indication of Hartmann’s reversal and time until the reversal of the procedure is summarized in Table 3. Exact number of studies contributing to each outcome is summarized in Table 2S in supplementary files.

**Table 2:** Characteristics of included studies

Studies characteristics					
Study	Year	Country	Funding	Study design	Follow-up in months
Abueta	2022	Colombia	–	Retrospective cohort	–
Achkasov	2010	Russia	–	Retrospective cohort	–
Akmercan	2025	Turkey	–	Case series	>24
Akmercan	2024	Turkey	No	Retrospective cohort	–
Aquina	2016	USA	–	Retrospective cohort	–
Arkenbosch	2015	USA	–	Retrospective cohort	–
Bottger	2011	Germany	–	Case series	–
Brathwaite	2015	USA	–	Retrospective cohort	–
Carus	2008	Germany	–	Case series	–
Caselli	2010	Chile	–	Case series	34
Cassini	2017	Italy	No	Retrospective cohort	6
Cellini	2013	USA	–	Retrospective cohort	–
Chen	2021	United Kingdom	No	Retrospective cohort	–
Cho	2021	Republic of Korea	–	Retrospective cohort	11.5
Choi	2015	Republic of Korea	No	Case series	–
Chouillard	2007	France	–	Case series	–
Clermonts	2016	USA	no	Prospective cohort	–
D’Alessandro	2020	France	–	Case–controlled study	12
De’Angelis	2013	France		Retrospective cohort	36
Faure	2007	France	–	Prospective cohort	20
Fiscon	2014	Italy	No	Case series	44
Froehlich	2025	USA	No	Case series	–
Giuliani	2020	Italy	–	Case series	–
Giuseppe	2019	Italy	–	Case series	36
Golash	2006	Oman	–	Case series	36
Goncharov	2023	Russia		Retrospective nonrandomized study	–
Haughn	2008	USA	–	Retrospective cohort	6
Horesh	2018	Israel	No	Retrospective study	–
Kang	2021	Korea	–	Case series	–
Katsura	2024	USA	No	Retrospective cohort	–
Kwak	2018	Korea	–	Retrospective study	–
Lee	2023	Korea	–	Retrospective cohort	–
Leroy	2011	France	–	Case series	–
Maitra	2013	United Kingdom	No	Case–controlled study	27
Mazeh	2009	Israel	–	Retrospective cohort	–
Melkonian	2017	Chile	No	Retrospective cohort	3
Minatti	2022	Argentina	–	Retrospective cohort	–
Mirza	2021	USA	No	Retrospective cohort	8.7 ± 6.8 months
Misra	2022	India	–	Retrospective cohort	–
NG	2013	China	–	Retrospective cohort	–
Nguyen	2022	Vietnam	No	Retrospective cohort	–
Onder	2016	USA	–	Retrospective cohort	–
Park	2018	Korea	–	Retrospective cohort	–
Pei	2018	USA	–	Retrospective cohort	–
Petersen	2009	Germany	–	Prospective cohort	–
Reali	2022	UK	–	Retrospective cohort	–
Rosen	2006	USA	–	Retrospective cohort	14.7
Sato	2022	Japan	–	Retrospective cohort	–
Steinemann	2015	Switzerland	–	Retrospective cohort	–
Svenningsen	2010	Denmark	–	Case series	–
Thambi	2019	UK	No	Retrospective cohort	–
Thambi	2021	UK	–	Case series	–
Vacher	2002	France	–	Case series	–
Van loon	2020	Netherlands	–	Case series	Minimum follow–up period consisted of 30 days postoperatively.
Walklett	2014	UK	–	Retrospective cohort	–
Whitny	2020	USA	–	Retrospective cohort	–
Yang	2014	Australia	–	Retrospective cohort	–
Zimmermann	2014	Germany	–	Retrospective cohort	Median follow–up of 8 months (range 1–20 months)
Imigo	2022	Chile	–	Retrospective cohort	–
Ottaviano	2023	USA	No	Retrospective cohort	–
Brathwaite	2017	USA	Yes	Retrospective cohort	–
Ferrari	2024	USA	–	Retrospective cohort	30–day follow–up
Tan	2023	China	–	Retrospective cohort	–
Manigrasso	2026	Italy	–	Retrospective cohort	–

**Table 3:** Patients characteristics

Patients’ characteristics										
Study	Number of patients (Laparoscopic or robotic)	Age (mean)	Males *n* (%)	BMI	Classification	Comorbidities	Indication of Hartmann’s procedure	History of previous operations	Time to reversal	Rectal stump length
Abueta	264	50 (18) median (IQR)	140 (53.0)	<18.5: 12 18.5–24.9: 92 25–29.9: 114 30–34.9: 39 35–39.9: 5 >40: 2	ASA Classification 1: 36 2: 147 3: 78 4: 3	Hypertension: 97 Coronary artery disease: 15 pulmonary disease: 19 Diabetes type 2: 30 immunosupression: 7 Congestive heart failure: 18 Anticoagulant: 18 Others: 72 None: 35	Cancer: 54 No cancer: 197 Trauma: 13	Prior abdominal surgery 1: 169 2: 52 3: 29 4: 9	–	–
Achkasov	36	55.7 (11.5)	19 (52)	28.6 (5.7)			Colon cancer: 22 Diverticulitis: 11 Abdomen trauma: 2 Villous tumor: 1 Endometriosis: 0 Tubeovarial abscess: 0	Cardiac surgery: 26	12.7 (7.4) months	23.7 (8.6)
Akmercan	23	62 (46–68)	14 (6.9)	26.4 (25.6–31.6)	ASA–PS score 1: 8 2: 13 3: 2	Diabetes mellitus: 9 Hypertension: 3 Heart disease: 2 Lung disease: 2	Diverticulitis: 7 Adenocarcinoma: 14 Trauma: 2	7	11 months	20 (15–30)
Akmercan	23	62 (30–81)	9 (39)	26.4 (21.3–38.1)	ASA score 1: 8 2: 13 3: 2		Tumor: 14 Diverticulitis: 7 Trauma: 2	7	11 months	20 (6–40)
Aquina	669	Not reported	Not reported	Not reported	Not reported	Not reported	Not reported	Not reported	Not reported	Not reported
Arkenbosch	732	59 (median)	415	27.6 (6.6)	ASA classification No disturb: 33 Mild disturb: 407 Severe disturb: 292	Diabetes: 63 Alcohol use: 18 Dyspnea: 41 FHS–partially dependent: 18 COPD: 37 Hypertension: 343 History of peripheral vascular disease: 5 Dialysis: 9 Choronic steroid use34 Weight loss: 17 Bleeding disorder: 22	–			
Bottger	15	61 (35–83)	5		ASA Classification 2: 3 3: 7 4: 5		In 13 out of 15 owing to peritonitis caused by perforated diverticulitis		Between 4 weeks and 6 months after primary operations	–
Brathwaite	19	56.6 (35–83)		28.5 (21–40)	ASA 2: 5 3: 12 4: 2	Hypertension 11 Gastroesophageal reflux disease 1 Tobacco use 12 Chronic obstructive pulmonary disease 2 Hyperlipidaemia 2 Diabetes 0 Obstructive sleep apnoea 1 Heart disease 5 Kidney disease 1 Liver dysfunction 0 Cancer 1 Alcoholism 1	Diverticulitis: 12 Trauma: 1 Ischemic colitis: 1 Other: 4	Prior abdominal surgery: 1.2	7.9	
Carus	28									
Caselli	30	61.5 (13)		26.1 (2)	1.8 (0.3)		Acute complicated diverticulitis 19 Obstructive neoplasms of the sigmoid colon 3 Perforated neoplasms of the sigmoid colon 2 Ischaemic colitis 2 Sigmoid volvulus 1 Iatrogenesis 1 Ischaemic colitis 1 Gynecological neoplasia 1	–		
Cassini	46	68.4 (30–85)	10 (21.7)	25.4 (19.2–27.6)	ASA classification 1: 6 2: 34 3: 6 Zulches’ class 1: 4 2: 8 3: 22 4: 12				11.4 weeks	
Cellini	336	59.5 (14.8)	184 (54)	≤ 30 is 95	ASA grade 3 and 4 is 125	Diabetes: 25 Smoker: 80 Functional status-dependent: 8 COPD: 20 Cardiac co–morbidity: 34 Renal insufficiency: 2 Neurological co–morbidities: 16 Steroid for chronic condition: 15 Bleeding disorders: 9	–			
Chen	22	58.8 (13.4)	13 (59)		ASA score 1: 2 2: 15 3: 5	Diabetes: 6 Ischemic heart disease: 2 Hypertension: 6	COLON cancer: 8 diverticulitis: 10 Perforation: 1 Trauma: 0 Others: 3			
Cho	20	69.6 (12.2)	7 (35%)	24.4 (3.1)	ASA classification 1: 0 2: 8 3: 12		Diverticulitis: 6 Ischemic colitis: 9 Faecal impaction: 1 Sigmoid colon cancer: 1 Rectosigmoid junction cancer: 2 Placental trophoblastic tumor: 1	–	3	
Choi	22	62 (21–87)	15 (68%)	22.2 (14.8–29.2)	ASA classification 1: 5 2: 14 3: 3 4: 0		Complicated diverticulitis: 6 Colorectal cancer: 6 Sigmoid volvulus: 4 Anastomosis insufficiency: 2 Ischemic colitis: 1 Others: 3	–	5.4	More than 10 cm in 15 patients
Chouillard	27								7	
Clermonts	25	52.2 (13.2)	18 (72)	27.8	ASA classification 1: 8 2: 15 3: 2		Diverticulitis: 15 Carcinoma: 7 Anastomotic dehiscence: 3	–	15.9 (7.9)	
D’Alessandro	SP–HR: 44 MP–HR: 44	SP–HR: 62 (12.9) MP–HR: 59 (13.8)	SP–HR: 30 MP–HR: 30	SP–HR: 24.2 (5.5) MP–HR: 23.2 (4.9)	ASA score SP–HR: 1: 14 2: 26 3: 4 4: 0 MP–HR: 1: 12 2: 27 3: 5 4: 0		SP–HR: Begnin/malignant: 29/15 MP–HR: Begnin/malignant: 30/14	–	SP–HR: 4.4 (2.8) MP–HR: 5.8 (3.8)	–
De’Angelis	28	54.9 (15.4)	12 (42%)	24.1 (3.1)	ASA classification 1: 16 2: 10 3: 2	Diabetes: 3 Cardiovascular: 9 Pulmonary disease: 2	Episodes of acute Diverticulitis before HP None: 16 One: 8 Two: 4	–	4.6	
Faure	14	61 (35–86)	6 (42%)		ASA score 1: 1 2: 11 3: 3		Diverticula: 9 CANCER: 4 colonic ischemia: 1		6 months	
Fiscon	3						Perforated diverticulitis: 12 Perforated colon cancer: 3 Other: 5	–	6 months	
Froehlich	37	57.1	21 (56.8%)	28.6 (21.4–48.7)	ASA class 2: 16 3: 21	Hypertension 17 (40.5%) Hyperlipidaemia 13 (35.1%) DMII 2 (5.4%) Coronary artery disease 0 (0.0%) Congestive heart failure 1 (2.7%) Chronic kidney disease 2 (5.4%) Chronic obstructive pulmonary disorder 2 (5.4%) Obstructive sleep apnea 5 (13.5%) History of CVA/stroke 1 (2.7%) Psychiatric history (anxiety, depression, bipolar, etc.) 14 (37.8%) Chronic steroid use 2 (5.4%) Preoperative biologic use within past 2 months 4 (10.8%) Smoking history Current 4 (10.8%) Former 12 (32.4%) Never 21 (56.8%) Performance status Independent 31 (97.3%) Partially dependent 1 (2.7%) Fully dependent 0 (0.0%)	Perforated diverticulitis 24 (64.9%) Non–diverticular perforations 4 (10.8%) Perforated stercoral colitis 1 Perforated colon malignancies 2 Perforation from acute Crohn’s flare 1 Complicated diverticulitis without frank perforation 3 (8.1%) Sigmoid stricture 1 Colovesical fistula 1 Undrainable abscess with failed improvement on antibiotics 1 Obstructing colon malignancy 1 (2.7%) Rectal invasion from gynecologic malignancy 1 (2.7%) Crohn’s colitis 1 (2.7%) Traumatic colon injuries 2 (5.4%) Fecal diversion for perineal wound 1 (2.7%)	–	Mean days: 286 Median days: 167 Range: 85–2121	–
Giuliani	24	69		26	ASA class 2: 14 3: 10	42% had comorbidities (mean Charlson comorbidity index 3)	acute complicated diverticulitis: 14 Obstructing sigmoid cancer: 6 Iatrogenic rectal perforation: 1		9 months (4–19 months)	–
Giuseppe	20	66 (44–87)	10 (50%)	<20: 1 20–25: 9 25–30: 5 >30: 3	ASA score 1: 2 2: 10 3: 8		Diverticulitis: 10 Tumor: 7 Trauma: 2 Volvulus: 1		Avg (days) 201 (range, 80–666)	–
Golash	12	40	8 (66%)				Perforated diverticular with intra–abdominal sepsis: 6 Traumatic rupture of left colon and rectum following RTA: 4 Stricture left colon at site of previous anastomosis: 1 Obstructed cancer of left colon: 1	–	Mean of 130 days (70–220 days)	–
Goncharov	19	56.6 (13.7)	7 (36.8%)	29 (4)	ASA score 1–2: 17 3: 2	Comorbidity index: 2.4	–		Mean of 4 months (1–9 months)	–
Haughn	61	59.2 (14.2)	27 (44%)	29.7 (8.5)	ASA grade: 1.6 (0.5)	–	Diverticulitis: 44 Cancer: 10 trauma: 7	–	5.7 (2)	–
Horesh	76	61.3 (24–89)	40 (52.6%)	26.6 (16.9–36.4)	–	–	Diverticular disease 38 (50%) Malignant obstruction 19 (25%) Benign obstruction 3 (3.9%) Perforation following colonoscopy 4 (5.2%) Anastomotic leak following colonic resection “Discussion” (5.2%) Trauma 3 (3.9%) Inflammatory bowel disease 1 (1.3%) Ischemic colitis 0 Infective colitis 0 Other 4 (5.2%)	–	6.83 (1.5–33.66)	–
Kang	20	61 (40–88)	8 (40%)	25.8 (20.1–38.9)	ASA PS classification) 1/2: 17 3/4: 3	–	Diverticulitis perforation: 8 Cancer perforation: 6 Cancer obstruction: 4 Others: 2	–	243 (day) (111–3790)	–
Katsura	laparoscopic: 3245 robotic: 1135	laparoscopic: 60 (51–69) robotic: 63 (52–71)	laparoscopic: 1755 (54%) robotic: 605 (53%)	BMI>=30 laparoscopic: 565 robotic: 185	–	–	–	–	–	–
Kwak	17	62.5 (15.8)	11 (64.7%)	24 (3.3)		–	Sigmoid colon perforation: 7 Sigmoid colon obstruction: 2 Anastomosis leakage: 6 Ischemic changes: 1 Rectal cancer: 1	–	528.7 (75–4417)days	–
Lee	63	70 (IQR62–75)	30 (47.62)	23.3 (IQR 20.9–25.8)	ASA classification: 1: 7 2: 47 3: 9	Diabates: 10 Hypertension: 39 Heart disease: 12 Pulmonary disease: 6 Liver disease: 1 Cerebrovascular disease: 7 Cancer or benign: 22	–	–	–	15cm (IQR 10–20)
Leroy	42	Male: 59.7 Female: 65.8	27 (64.3%)	<30: 33 >30: 9	ASA classification: 1: 10 2: 20 3: 12		Sigmoid diverticulitis: 32 Perforation during colonoscopy: 5 Cancer: 3 Colorectal anastomosis leakage: 1 Volvulus: 1	–	SFM: 124 Non–SFM: 137	–
Maitra	45	59	26 (57%)	28.4	–	–	Diverticular disease: 38 Stercoral perforations: 2 Ischemic bowel: 2 Cancer complications: 2 Perineal trauma: 1	–	–	–
Mazeh	41	58.49 (33–85)	20 (48%)	26.78 (19.5–40.8)	–	No. of comorbidities: 1.58 (0–6)	Diverticulitis: 24 Perforation: 9 Colorectal carcinoma: 2 Other: 6	No. of previous surgeries: 1.45 (1–4)	148.73 (41–962)	–
Melkonian	49 laparoscopic	55years (25–90)	30	–	ASA score<= 2 (Most of the patients)	–	Diverticulitis 32 (65%) Colorectal carcinoma 6 (12%) Sigmoid volvulus 6 (12%) Trauma — Other 5 (10%)	–	–	–
Minatti	62	58 years (range 17 to 95)	–	–	ASA *I* 45% (10/22) ASA II 29% (12/41) ASA III 66% (4/6)	–	–	–	9 months	–
Mirza	33 laparoscopic	51.5 (25−19)	23 (69.7)	28.1 ± 4.9	2.4 ± 0.6	20 (60.6)	Diverticulitis 18 (54.6) Trauma 5 (15.2) Colorectal cancer 8 (24.2) Inflammatory bowel disease 2 (6.1) Colitis 2 (6.1) Other 1 (3)	5 (15.2) (More than one pelviabdominal surgery)	–	–
Misra	32	(mean 47.5 years).	12 (37.5)	–	–	–	–	–	–	–
NG	47 laparoscopic	61 (range: 34–84)	–	–	–	–	Sigmoid volvulus 0 Perforated diverticulitis 13 Other perforations 3 Colonic malignancy 27 Gynecological malignancy 4 Anastomotic leakage 0	–	–	–
Nguyen	35 laparoscopic	61 (20–78)	21	–	ASA PS classification *I* 3 (8.6) II 26 (74.3) III 6 (17.1)	–	Rupture of colon cancer 5 (14.3) Perforated diverticulitis 12 (34.3) Obstructed colorectal cancer 14 (40.0) Trauma 4 (11.4)	–	–	–
Onder	18 laparoscopic	50.8±16.7 (21–77)	10	28.6±6.5 (21–45)	ASA II 5 (27.8) III 13 (72.2) IV 0 (0)	15 (83.3)	–	4 (25)	6.3±6.9 (4–34)	–
Park	20 laparoscopic	63.7 ± 12.4	13	23.3 ± 2.5	2.55 ± 1.6	Diabetes 3 hypertension 10	Colon cancer 12 (60) Diverticular perforation 2 (10) Stercoral colitis 3 (15) Traumatic colon injury 2 (10) Ischemic colitis 1 (5) Postoperative adhesion – Sigmoid volvulus – Total 20	–	–	–
Pei	2423 laparoscopic	57.66 ±15.14	1223 (50.47)	–	–	609	Neoplasm,98 Diverticular disease, 157 Colostomy complications, 108 Colostomy status, 1122 Others, 938	–	–	–
Petersen	71 laparoscopic	–	39 (54.9)	–	–	–	–	–	–	–
Reali	35 laparoscopic	61.1 (48.2–73.3)	20 (57)	–	*I* 4 II 26 III 5	–	Indication Cancer 9 Diverticulitis 19 Crohn’s disease 0 Iatrogenic 5 Trauma 1 Volvulus 0 Thrombosis 1 0	–	–	Long 30 Short 5
Rosen 2005	22 laparoscopic	54 years (range 33–73 years)	–	–	–	–	Perforated sigmoid diverticulitis 15 Perforated colon cancer 2 Fournier’s gangrene 2 Anastomotic dehiscence 1 Traumatic colonic injury 1 Severe radiation colitis 1		5.5 (2.3–12.6) months	–
Rosen 2006	22 laparoscopic	54 years (range 33–73 years)	–	–	–	–	Perforated sigmoid diverticulitis 15 Perforated colon cancer 2 Fournier’s gangrene 2 Anastomotic dehiscence 1 Traumatic colonic injury 1 Severe radiation colitis 1		5.5 (2.3–12.6) months	–
Sato	19 laparoscopic	66 (32–81)	11 (57.9)	–	ASA–*I* 2 (10.5) ASA–II 15 (78.9) ASA–III 2 (10.5)	Diabetes mellitus,4 (21.1) Hypertension, 7 (36.8) Acute coronary disease, 0 (0) Respiratory disease, 1 (6.7) Steroid user, 2 (10.5) Chronic kidney disease, 2 (10.5)	Diverticulitis with perforation, 9 (47.4) Colorectal cancer with obstruction, 3 (15.8) Colorectal cancer with perforation,2 (10.5) Traumatic colorectal perforation, 2 (10.5) Colonic necrosis, 1 (5.3) Anastomotic leakage, 0 (0) Colonic perforation by inguinal hernia, 0 (0) Stercoral perforation of colon, 1 (5.3) Rectal injury during gynecological operation,1 (5.3)	–	7.9 (3.4–31.1) months	Colon, 4 (21.1) Upper rectum, 12 (63.2) Lower rectum, 3 (15.8)
Steinemann	32 laparoscopic	75 (67–83)	15 (46.8)	26 (22–31)	ASA Status, n/N (%) ASA *I*, 0/32 (0 %) ASA II, 6/32 (18.3 %) ASA III, 16/32 (50 %) ASA IV, 10/32 (31.3 %) ASA ≥ III Total, 26/32 (81.3 %)	–	Diverticulitis,21/32 (65.7 %) Other perforation, 4/32 (12.5 %) Anastomotic leakage, 4/32 (15.2 %) Colon ischemia, 3/32 (9.4 %)	–	3.7 (2.8–4.4) months	–
Svenningsen	21 laparoscopic	61 (26–79)	13 (62%)	23 (19–31)	ASA score, median (range) 2 (1–2)	–	Iatrogenic perforation 4 Cancer obstruction 3 Diverticulitis 13 Gynaecologic disease 1	–	5.9 (3–12.8) months	20 (12–40) median and range, cm
Thambi 2019	56 laparoscopic	62 (32–87)	34 (60.7)	29 (21–42)	ASA *I* 8 ASA II 28 ASA III 19 ASA IV 1	–	–	1	–	–
Thambi 2021	68 laparoscopic	62 [33–88]	39 (57%)	29.4 [20.5–42.2]	ASA *I* 08 ASA II 37 ASA III 22 ASAIV 01	Hypertension 29 Diabetes 08 COPD 12 Renal Disease 04 Cardiac illness 18	Anastomotic leak 01 Diverticular complications 46 Malignancy 16 Miscellaneous 05	14	–	–
vacher	38 laparoscopic	60±13.5 years		–	–	–	Sigmoid diverticulitis, 26 Colonic perforation, 6 Colonic neoplasm with obstruction, 2 Sigmoid volvulus, 2 Ischemic colitis, 1, Ulcerative colitis, 1	–	–	–
Van loon	72 laparoscopic	–	–	–	–	–	–	–	–	–
Walklett	12 laparoscopic	–	–	–	–	–	–	–	–	–
Whitny	70 laparoscopic	61.8 (16.5)	42 (60)	–	ASA IV 4 (6.4)	–	Diverticular disease, 53 (75.7) Malignancy of colon or rectum, 3 (4.3) Volvulus, 1 (1.4) Ischemia, 1 (1.4) Anastomotic leak, 3 (4.3) Other, 7 (10.0) Emergent HP, 48 (71.6)	–	6.3 months (± 5.1)	–
Yang	43 laparoscopic	60.9 ± 14.4	26 (60)	29.7 ± 6.5	*I* 9 (27%) II 19 (56%) III 6 (18%)	–	Colon cancer, 5 (12%) Complications of diverticular disease, 26 (61%) • Bleeding, 1 • Obstruction, 2 • Perforation, 17 • Diverticulitis, 4 • Recurrent diverticulitis, 2 Iatrogenic injury, 3 (7%) Other, 5 (12%)	–	–	–
Zimmermann	24 laparoscopic	46 (27–84)	14	26.5 (19.5–34.9	*I* 2 (8.3%) II 13 (54.2%) III 9 (37.5%) IV 0	–	Sigmoid diverticulitis 15 (62.5%) Malignant disease 1 (4.2%) Ischemic event 3 (12.5%) Iatrogenic perforation 1 (4.2%) M. Crohn perforation 3 (12.5%) Anastomosis insufficiency 0 Radiation stenosis 0 Other 1 (4.2%)	Necrotizing pancreatitis 0 Anal fistula 1 (4.1%) Appendectomy 2 (8.2%) Other 3 (12.5%)	3 months (5.5–8)	–
Imigo	41 laparoscopic	62.4 (14.7)	21	25.9 (4)	*I* 8 II 28 III 5	Comorbidity (overall) 32 (78) Hypertension 21 (51.2) Diabetes 3 (7.3) Pulmonary disease 1 (2.4) Heart disease 5 (12.2) Immunosuppression 5 (12.2) Noncolorectal cancer 3 (7.3) Smoking 6 (6.9) Anticoagulant therapy 2 (4.9) Previous chemotherapy 8 (19.5) Parastomal hernia 1 (2.4)	Diverticular disease 21 (51.2) Colorectal cancer 10 (24.4) Leak / Fistula 2 (4.9) Iatrogenic injury 2 (4.9) Sigmoid volvulus 1 (2.4)	–	6 (3–23)	–
Ottaviano	1274	Not reported	Not reported	Not reported	Not reported	Not reported	Not reported	Not reported	Not reported	Not reported
Brathwaite	1431 laparoscopic	57.4 (18–89)	724 (51)	27.4 (11.5–72.6)	*I* 58 (4) II 799 (56) III 538 (38) IV 36 (3) V 0 (0)	Diabetes 146 (10) Tobacco use 304 (21) Steroid use 66 (5)	–	–	–	–
Ferrari	48 laparoscopic	59.3 (13.1)	15 (31.3%)	30.3 (6.9)	II 24 (50.0%) III 22 (45.8%) IV 2 (4.2%)	Diabetes 7 (14.6%) Steroids 11 (22.9%) Immunosuppression 4 (8.3%) CVS disease 14 (29.2%) CKD 9 (18.8%) Smoker currently 3 (6.3%)	Diverticulitis 28 (58.3%) Cancer 3 (6.3%) Other 17 (35.4%)	–	12.8 (18.7) months	–
Tan	48 laparoscopic	59.9 ± 13.0	28 (58.3%)	23.6 ± 3.7	–	Smoking 21 (43.8%) Drinking 16 (33.3%) HTN 11 (22.9%) T2DM 2 (4.2%)	–	–	–	–
Manigrasso	Robotic: 45 Laparoscopic: 44	Robotic: 65.93 ± 12.97 Laparoscopic: 65.57 ± 11.91	Robotic: 19 (42.2) Laparoscopic: 22 (50)	Robotic: 25.74 ± 3.82 Laparoscopic: 26.13 ± 4.29	ASA Score Robotic: *I* 1 (2.2) II 32 (71.1) III 12 (26.7) IV 0 (0) Laparoscopic: *I* 0 (0) II 32 (72.7) III 12 (27.3) IV 0 (0)	–	Robotic: Tumour 13 (28.9) Diverticulitis 32 (71.1) Laparoscopic: Tumour 7 (15.9) Diverticulitis 37 (84.1)	Robotic: 15 (33.3) Laparoscopic: 23 (52.3)	–	–

### Intraoperative outcomes

**Fig. 2:**
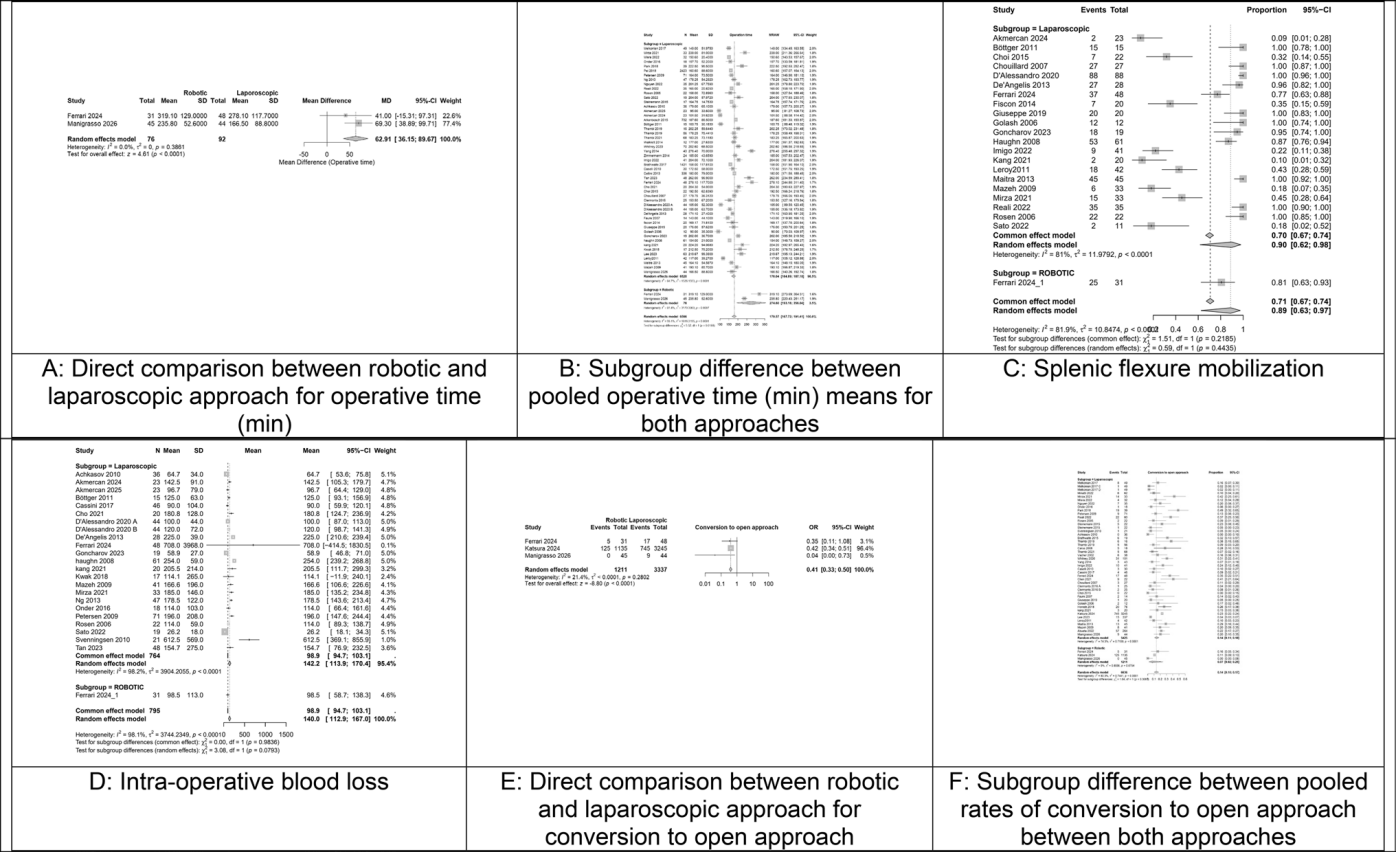
Intraoperative outcomes

Intraoperative outcomes included operation time, splenic flexure mobilization, intraoperative blood loss, and conversion to open approach. Direct comparative analysis for operation time (min) showed a significantly higher mean difference (MD) in robotic approach (MD 62.91 [95% CI 36.15, 89.67]; I^2^ 0%; *P*< 0.001) (Fig. 2A). The pooled mean for operation time (min) for laparoscopic approach was 176.04 (I^2^ = 94.7%), while the robotic approach was 274.6 (*I*^2^ = 90.4%). Subgroup analysis demonstrated a statistically significant difference in operation time between both approaches (*P* = 0.018) (Fig. 2B). On the other hand, the pooled proportions for splenic flexure mobilization were 90% (*I*^2^ = 81%) and 81% (I^2^ = N/A) respectively, the subgroup difference did not yield statistical significance (*P* = 0.4435) (Fig. 2C). Additionally, intraoperative blood loss pooled mean was 142.2 (*I*^2^ = 98.2%) and 98.5 (*I*^2^ = N/A), respectively, with a nonsignificant subgroup difference (*P*= 0.0793) (Fig. 2D).

Furthermore, direct comparative analysis for conversion to open approach demonstrated a statistically significant smaller odds ratio (OR) in robotic approach (OR 0.41 [95% CI 0.33, 0.50]; I^2^ 21.4%; *P*< 0.001) (Fig. 2E).However, pooled proportions for conversion to open approach were 14% (I^2^ = 74.3%) and 7% (I^2^ = 0%), respectively; however, the subgroup difference was not statistically significant (*P*= 0.3) (Fig. 2F).

### Short-term postoperative outcomes

**Fig. 3:**
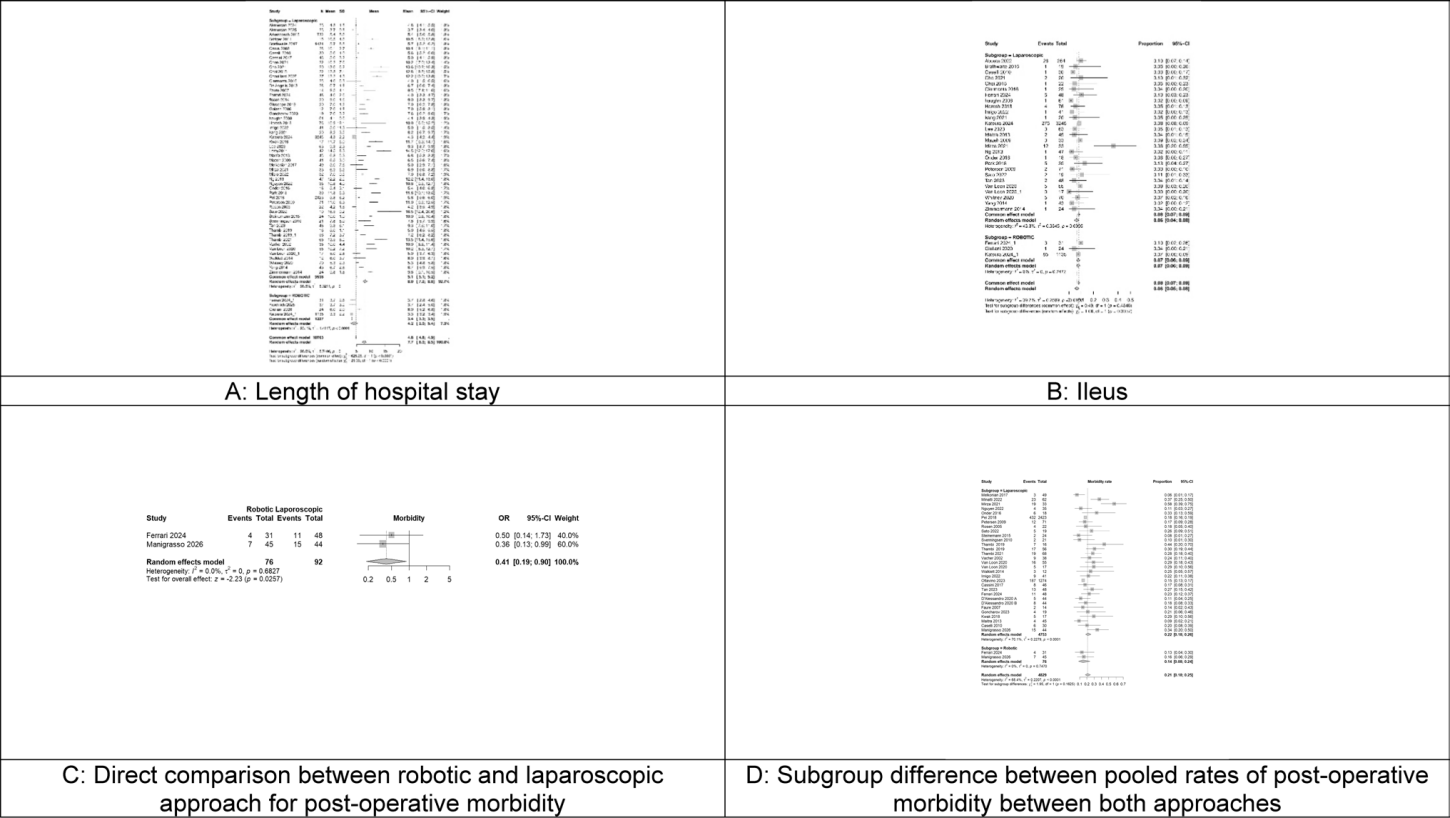
Short-term postoperative outcomes

Length of hospital stay pooled mean was 8 (*I*^2^ = 98.5%) and 4.2 (*I*^2^ = 93.1%), respectively, with a statistically significant subgroup difference (*P* < 0.001) (Fig. 3A). On the contrary, pooled proportions for ileus were 6% (*I*^2^ = 43.3%) and 7% (*I*^2^ = 0%) respectively; however, the subgroup difference did not reach statistical significance (*P* = 0.3037) (Fig. 3B). Direct comparative analysis for postoperative morbidity demonstrated a statistically significant smaller odds ratio (OR) in robotic approach (OR 0.41 [95% CI 0.19, 0.90]; *I*^2^ 0%; *P* = 0.025) (Fig. 3C). Morbidity in the laparoscopic approach showed a pooled proportion of 22% (*I*^2^ = 70.1%), while in the robotic approach showed 14% (*I*^2^ = 0%). Nevertheless, the subgroup difference was statistically insignificant (*P* = 0.16) (Fig. 3D).

**Fig. 4:**
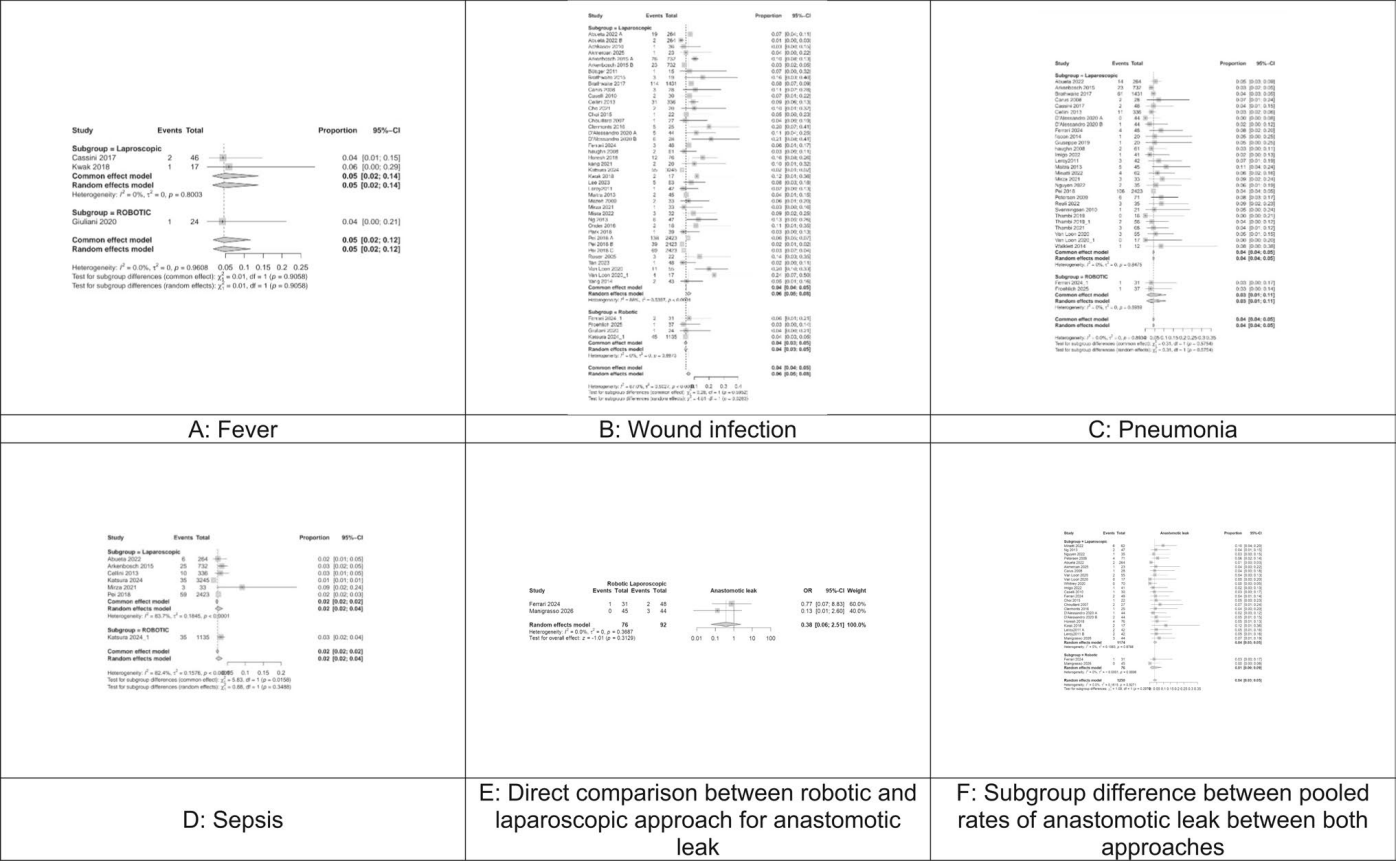
Continued short-term postoperative outcomes

Fever pooled proportions were 5% (*I*^2^ = 0%) and 4% (*I*^2^ = N/A), respectively, with insignificant subgroup difference (*P* = 0.9058) (Fig. 4A). However, surgical site infection pooled proportions showed 6% (*I*^2^ = 88%) and 4% (*I*^2^ = 0%) respectively, with a statistically significant subgroup difference (*P *= 0.0283) (Fig. 4B). Surprisingly, pneumonia showed pooled proportions of 2% (*I*^2^ = 43.6%) and 5% (*I*^2^ = 0%), respectively, with a statistically significant subgroup difference (*P* < 0.001) (Fig. 4C). On the contrary, sepsis pooled proportions were 2% (*I*^2^ = 83.7%) and 3% (*I*^2^ = N/A), respectively; however, the subgroup difference was not statistically significant (*P* = 0.3488) (Fig. 4D).

Direct comparative analysis anastomotic leakage demonstrated a smaller odds ratio (OR) in robotic approach; however, the ratio did not reach statistical significance (OR 0.38 [95% CI 0.06, 2.51]; *I*^2^ 0%; *P* = 0.312) (Fig. 4E).Pooled proportions for anastomotic leak showed 4% (*I*^2^ = 0%) and 1% (*I*^2^ = 0%), respectively, though subgroup difference was insignificant (*P* = 0.29) (Fig. 4F).

### Long-term postoperative outcomes

**Fig. 5:**
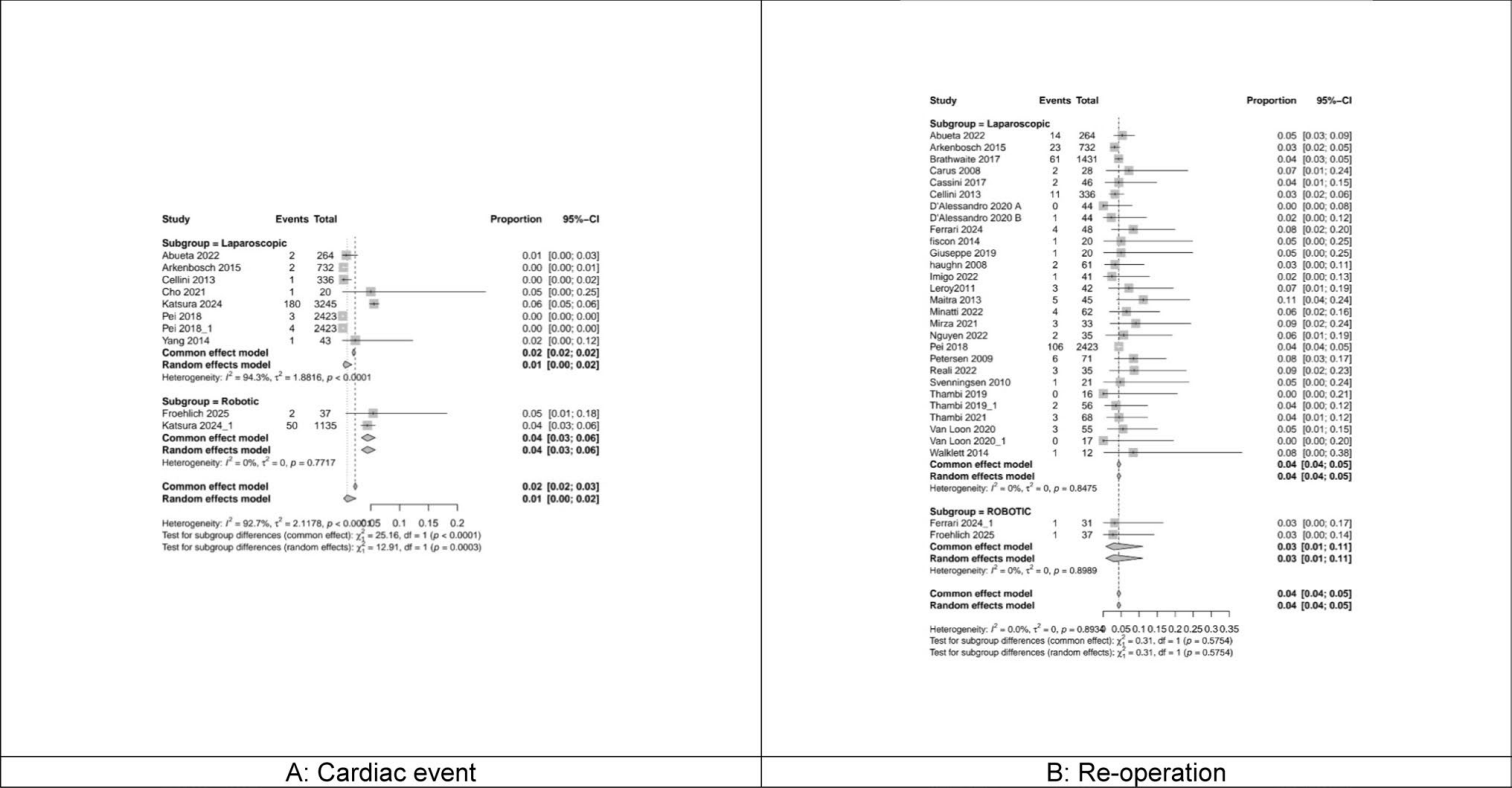
Long-term postoperative outcomes

Pooled proportions for cardiac events were 1% (*I*^2^ = 94.3%) and 4% (*I*^2^ = 0%), respectively, with a statistically significant subgroup difference (*P* = 0.0003) (Fig. 5A**).** Additionally, reoperation pooled proportions were 4% (*I*^2^ = 0%) and 3% (*I*^2^ = 0%), respectively, with statistically insignificant subgroup difference (*P* = 0.5) (Fig. 5B).

### Sensitivity analysis (leave one out) and publication bias assessment

Owing to observed heterogeneity across studies, sensitivity analysis was performed for all outcomes. Exclusion of Mirza 2021 [62] resulted in complete heterogeneity resolution for ileus (*I*^2^ = 0%). Additionally, omission of Katsura 2024 [7] reduced heterogeneity for pneumonia and sepsis to 39.5% and 28.6%, respectively. However, leave-one-out sensitivity analyses for the remaining outcomes confirmed the robustness of the pooled estimates, indicating that no single study exerted a disproportionate influence on the overall effect (Figs. 1S, 14S in Supplementary files).

Visual inspection of the funnel plots suggested asymmetry for most outcomes, except for morbidity. However, Egger’s regression test demonstrated statistically significant effects only for operative time, conversion to an open approach, length of hospital stay, anastomotic leak, and cardiac events (*P* = 0.0189, *P* = 0.028, *P* < 0.001, *P* = 0.0072, and *P* = 0.0154, respectively). However, these findings should be interpreted cautiously, as several outcomes were based on fewer than ten studies, limiting the statistical power and reliability of funnel plots and Egger’s regression test (Figs. 16S, 28S in Supplementary files)

### AMSTAR 2 checklist

The methodological integrity of this systematic review was evaluated using the AMSTAR 2 checklist; the review satisfied most AMSTAR 2 domains, supporting overall methodological rigor. On the basis of commonly used thresholds, adherence above 70% indicates strong methodological quality. According to AMSTAR 2 guidance, this classifies our review as high confidence, suggesting that its findings are reliable and robust (Table 3S in supplementary files).

### GRADE assessment

Using the GRADE framework, the certainty of evidence ranged from low to very low across all outcomes. All outcomes were downgraded because the available evidence was derived exclusively from observational studies. In addition, indirect comparisons between robotic and laparoscopic approaches, substantial heterogeneity for several outcomes, imprecision resulting from the limited number of robotic studies, and suspected publication bias further reduced the certainty of evidence for selected outcomes (Table 4).

**Table 4:** GRADE assessment

Outcome	Risk of bias	Indirectness	Inconsistency	Imprecision	Publication bias	Overall certainty
Operative time	Serious	Serious	Serious	Not serious	Suspected	Very low
Splenic flexure mobilization	Serious	Serious	Serious	Serious	Undetected	Very low
Blood loss	Serious	Serious	Serious	Not serious	Suspected	Very low
Conversion to open surgery	Serious	Serious	Serious	Serious	Suspected	Very low
Length of hospital stay	Serious	Serious	Serious	Not serious	Suspected	Very low
Ileus	Serious	Serious	Not serious	Serious	Undetected	Low
Morbidity	Serious	Serious	Not serious	Not serious	Undetected	Low
Fever	Serious	Serious	Not serious	Serious	Undetected	Low
Surgical site infection	Serious	Serious	Serious	Not serious	Undetected	Low
Pneumonia	Serious	Serious	Not serious	Serious	Undetected	Low
Sepsis	Serious	Serious	Serious	Serious	Undetected	Very low
Anastomotic leak	Serious	Serious	Not serious	Serious	Suspected	Very low
Cardiac events	Serious	Serious	Serious	Serious	Suspected	Very low
Reoperation	Serious	Serious	Not serious	Serious	Undetected	Low

## Discussion

Our meta-analysis aimed to compare perioperative outcomes between laparoscopic and robotic-assisted Hartmann’s reversal; however, interpretation of these findings requires caution because the available robotic evidence remains limited. Differences observed between robotic and laparoscopic subgroups represent indirect comparisons; consequently it might be influenced by differences in patient selection, institutional expertise, case complexity, and study design. It is considered one of the most technically demanding restorative colorectal operations, as it is often performed in a hostile abdomen with dense adhesions, distorted anatomy, and limited pelvic working space, often in patients with a substantial comorbidity burden [87].

Across included studies, robotic Hartmann reversal was associated with longer operative duration than laparoscopy. This pattern is biologically and operationally plausible: robotic docking, trocar placement strategy, and instrument exchanges can add nontrivial time, particularly during early adoption phases [88]. Moreover, the robotic cohort may have been selectively enriched with more complex cases (e.g., hostile pelvis, severe adhesions, high body mass index (BMI), prior sepsis), which can prolong operative time independent of the surgical platform. This interpretation is supported by Katsura et al. (2024), who similarly reported longer operative times for robotic procedures in a complex restorative colorectal setting [7]. By contrast, Ferrari et al., 2026 reported comparable operative times with robotics, which may reflect high-volume teams, streamlined docking workflows, or a later learning-curve phase [6]. Clinically, longer operative time does not necessarily imply inferior quality; it may represent “time invested” in safer dissection, more careful adhesiolysis, or avoidance of conversion. However, it has implications for theater efficiency, staffing, and cost factors that are central to the value proposition of robotics in a procedure already known for high resource use [89].

The pooled subgroup analysis showed a shorter length of stay in the robotic group. This finding is consistent with the hypothesis that robotics may facilitate less traumatic tissue handling, more precise dissection, and potentially smoother postoperative recovery, especially when robotics helps avoid large incisions or extensive traction in dense adhesions [12]. Kleiman et al., 2020, reported earlier discharge after robotic restorative colorectal procedures [90]. Conversely, Giuliani et al., 2020 did not observe a difference, which may be explained by differences in ERAS implementation, discharge culture, stoma-care logistics, or social factors driving discharge timing [12]. Importantly, length of stay is a composite, system-sensitive endpoint: it reflects not only surgical stress but also postoperative pathways, pain control strategies, bowel function protocols, and institutional thresholds for discharge [91]. Therefore, the observed reduction suggests potential downstream value for robotics, but it should be interpreted in the context of care pathways and selection effects.

Our pooled subgroup analysis showed lower reported SSI rates in robotic studies. Milone et al. 2023 reported fewer wound complications with robotic restorative colorectal surgery, where improved precision may reduce dead space or hematoma formation [92]. Mechanistically, robotics could reduce SSI indirectly by decreasing the need to convert to open surgery and by enabling intracorporeal techniques (e.g., controlled specimen extraction, meticulous hemostasis, minimized traction) [12]. Still, SSI is strongly influenced by patient factors (diabetes, obesity, smoking), contamination class, operative duration, and perioperative bundles; the apparent benefit therefore supports, but does not prove, a causal platform effect [93].

An unexpected finding in our analysis was a higher pneumonia and cardiac events (postoperatively) rates in the robotic group. A plausible explanation is confounding by case selection: surgeons may preferentially assign higher-risk patients (e.g., older, higher ASA, higher BMI, complex adhesions requiring prolonged Trendelenburg) to robotic surgery to leverage its ergonomic and technical advantages [94]. Longer operative times and potentially more prolonged pneumoperitoneum/positioning may also contribute to postoperative pulmonary and cardiac complications in susceptible patients [80]. In contrast to our results, Giuliani et al. (2020) reported no difference in postoperative pulmonary and cardiac outcomes between platforms, possibly reflecting more standardized anesthesia-protective ventilation, aggressive early mobilization, and mature ERAS pathways [12]. Clinically, “platform selection” must be integrated with perioperative respiratory and cardiac risk stratification and optimization, e.g., prehabilitation, smoking cessation, protective ventilation, fluid strategy, early mobilization, particularly when prolonged positioning is anticipated [95].

Direct comparative meta-analysis, based on the three studies reporting both approaches within the same cohort, showed significantly lower conversion to open surgery and postoperative morbidity with robotics. Neither difference, however, was significant when the same outcomes were assessed as pooled single-arm proportions, likely reflecting the greater power of head-to-head comparison over indirect pooling of heterogeneous cohorts, together with possible selection of high-volume, experienced centers among the comparative studies. As only three studies contributed direct data, these findings should be considered preliminary pending confirmation in larger comparative cohorts.

Several outcomes did not demonstrate statistically meaningful differences between robotic and laparoscopic Hartmann reversal, including intraoperative blood loss, conversion to open surgery, postoperative ileus, anastomotic leak, and reoperation. Collectively, these neutral findings suggest that, within the limits of available comparative evidence, both MIS platforms can achieve comparable technical safety and major complication profiles for Hartmann reversal when performed by appropriately experienced teams [96]. It is also plausible that true differences exist but remain undetected owing to limited robotic sample size, event rarity (e.g., leaks), variability in definitions/reporting, and confounding by selection.

## Limitations

First, the robotic evidence base remains limited compared with laparoscopy, and most comparisons between the two approaches were indirect, resulting primarily from pooled single-arm analyses, increasing uncertainty and widening susceptibility to selection bias. Second, most included studies are retrospective and nonrandomized, limiting control of baseline risk differences. Third, learning-curve effects are particularly relevant for robotics and can distort operative time and complication rates depending on institutional maturity. Fourth, clinical heterogeneity is substantial; differences in ERAS implementation, stoma management, anastomotic technique, and use of diverting ileostomy can affect the pooled results.

Fifth, subgroup analyses were based on pooled study level estimates rather than on individual patient data; consequently, statistically significant subgroup differences should not be interpreted as evidence of a causal effect of the surgical approach due to their susceptibility to ecological bias and residual confounding. Moreover, the robotic literature was limited, more recent, and frequently derived from high-volume centers with greater minimally invasive experience. As a result, temporal trends, learning-curve effects, center-specific expertise, and differences in patient selection or case complexity may have contributed to the observed differences independently of the surgical platform.

Finally, cost and value endpoints were not meta-analyzable in our study due to insufficient data. Future studies should focus on direct comparisons between robotic and laparoscopic approaches through randomized controlled trials or prospective observational studies, while also systematically reporting direct costs, operating room utilization, and patient-centered recovery outcomes.

## Conclusions

This systematic review and meta-analysis suggests that robotic-assisted Hartmann reversal may be associated with longer operative duration, yet may confer shorter length of stay and lower surgical site infection compared with laparoscopy. Direct comparative meta-analysis additionally suggested lower conversion to open surgery and lower postoperative morbidity with robotics; however, neither outcome differed significantly when assessed as pooled single-arm proportions. By contrast, signals of higher pneumonia and cardiac events in robotic cohorts, particularly in older and more complex patients, may reflect baseline-risk imbalance and/or the physiological impact of prolonged operative time and positioning rather than definitive platform-related harm; nevertheless, these findings highlight the importance of careful patient selection, perioperative optimization, and a realistic appraisal of resource utilization, as robotics may require greater time and cost. For major technical safety endpoints, including conversion, blood loss, anastomotic leak, and reoperation, the available evidence indicates broadly comparable performance between approaches, although the limited robotic sample size constrains certainty.

Taken together, while robotics may offer incremental recovery and wound-related advantages in selected settings, laparoscopy remains a highly suitable default approach for Hartmann reversal in many institutions, and the robotic platform may be best reserved for appropriately selected complex cases, structured training contexts, and centers with established expertise and efficiency.

Ultimately, although the robotic approach may offer potential advantages in Hartmann reversal, comparable outcomes can be achieved when laparoscopic surgery is performed by experienced surgeons.

## Supplementary Information

Below is the link to the electronic supplementary material.

Supplementary file1 (DOCX 8015 KB)

## Data Availability

All data analyzed during this study are included in this published article and its supplementary information files.
