# Supplementary material for: Does robotics add value in Hartmann’s reversal? A PRISMA-based systematic review and meta-analysis of perioperative outcomes
Source: Tech Coloproctol. 2026 Aug 14;30(1):133. doi: 10.1007/s10151-026-03414-5 (PMC13486091; doi:10.1007/s10151-026-03414-5)
Supplement: Supplementary file 1 [file 10151_2026_3414_MOESM1_ESM.docx]

Figure 1S: Leave-one-analysis operation time

Figure 2S: Leave-one-analysis splenic flexure mobilization

Figure 3S: Leave-one-analysis blood loss

Figure 4S: Leave-one-analysis conversion to open approach

Figure 5S: Leave-one-analysis length of hospital stay

Figure 6S: Leave-one-analysis ileus

Figure 7S: Leave-one-analysis morbidity

Figure 8S: Leave-one-analysis anastomotic leak

Figure 9S: Leave-one-analysis reoperation

Figure 10S: Leave-one-analysis fever

Figure 11S: Leave-one-analysis surgical site infection

Figure 12S: Leave-one-analysis sepsis

Figure 13S: Leave-one-analysis pneumonia

Figure 14S: Leave-one-analysis cardiac event

Figure 15S: Funnel plot operation time

Figure 16S: Funnel plot splenic flexure mobilization

Figure 17S: Funnel plot blood loss

Figure 18S: Funnel plot conversion to open approach

Figure 19S: Funnel plot length of hospital stay

Figure 20S: Funnel plot ileus

Figure 21S: Funnel plot morbidity

Figure 22S: Funnel plot anastomotic leak

Figure 23S: Funnel plot reoperation

Figure 24S: Funnel plot fever

Figure 25S: Funnel plot surgical site infection

Figure 26S: Funnel plot sepsis

Figure 27S: Funnel plot pneumonia

Figure 28S: Funnel plot cardiac event

Figure 29S: Funnel plot Conversion to open approach

Figure 30S: Funnel plot direct comparison conversion to open approach

Figure 31S: Funnel plot anastomotic leak

Figure 32S: Funnel plot operative time

Figure 33S: Funnel plot morbidity

Figure 34S: Leave-one-analysis operative time

Figure 35S: Leave-one-analysis conversion to open approach

Figure 36S: Leave-one-analysis direct conversion to open approach

Figure 37S: Leave-one-analysis anastomotic leak

Figure 38S: Leave-one-analysis morbidity

| **Section and Topic** | **Item #** | **Checklist item** | **Location where item is reported** |
| --- | --- | --- | --- |
| **TITLE** | | |  |
| Title | 1 | Identify the report as a systematic review. | P3 |
| **ABSTRACT** | | | P3 |
| Abstract | 2 | See the PRISMA 2020 for Abstracts checklist. | P3 |
| **INTRODUCTION** | | |  |
| Rationale | 3 | Describe the rationale for the review in the context of existing knowledge. | P2 |
| Objectives | 4 | Provide an explicit statement of the objective(s) or question(s) the review addresses. | P2,3 |
| **METHODS** | | |  |
| Eligibility criteria | 5 | Specify the inclusion and exclusion criteria for the review and how studies were grouped for the syntheses. | P4 |
| Information sources | 6 | Specify all databases, registers, websites, organisations, reference lists and other sources searched or consulted to identify studies. Specify the date when each source was last searched or consulted. | P4,5 |
| Search strategy | 7 | Present the full search strategies for all databases, registers and websites, including any filters and limits used. | P4,5 |
| Selection process | 8 | Specify the methods used to decide whether a study met the inclusion criteria of the review, including how many reviewers screened each record and each report retrieved, whether they worked independently, and if applicable, details of automation tools used in the process. | P4,5 |
| Data collection process | 9 | Specify the methods used to collect data from reports, including how many reviewers collected data from each report, whether they worked independently, any processes for obtaining or confirming data from study investigators, and if applicable, details of automation tools used in the process. | P4,5 |
| Data items | 10a | List and define all outcomes for which data were sought. Specify whether all results that were compatible with each outcome domain in each study were sought (e.g. for all measures, time points, analyses), and if not, the methods used to decide which results to collect. | P4,5 |
|  | 10b | List and define all other variables for which data were sought (e.g. participant and intervention characteristics, funding sources). Describe any assumptions made about any missing or unclear information. | P4,5 |
| Study risk of bias assessment | 11 | Specify the methods used to assess risk of bias in the included studies, including details of the tool(s) used, how many reviewers assessed each study and whether they worked independently, and if applicable, details of automation tools used in the process. | P4,5 |
| Effect measures | 12 | Specify for each outcome the effect measure(s) (e.g. risk ratio, mean difference) used in the synthesis or presentation of results. | P4,5 |
| Synthesis methods | 13a | Describe the processes used to decide which studies were eligible for each synthesis (e.g. tabulating the study intervention characteristics and comparing against the planned groups for each synthesis (item #5)). | P4,5 |
|  | 13b | Describe any methods required to prepare the data for presentation or synthesis, such as handling of missing summary statistics, or data conversions. | P4,5 |
|  | 13c | Describe any methods used to tabulate or visually display results of individual studies and syntheses. | P4,5 |
|  | 13d | Describe any methods used to synthesize results and provide a rationale for the choice(s). If meta-analysis was performed, describe the model(s), method(s) to identify the presence and extent of statistical heterogeneity, and software package(s) used. | P4,5 |
|  | 13e | Describe any methods used to explore possible causes of heterogeneity among study results (e.g. subgroup analysis, meta-regression). | P4,5 |
|  | 13f | Describe any sensitivity analyses conducted to assess robustness of the synthesized results. | P4,5 |
| Reporting bias assessment | 14 | Describe any methods used to assess risk of bias due to missing results in a synthesis (arising from reporting biases). | P4,5 |
| Certainty assessment | 15 | Describe any methods used to assess certainty (or confidence) in the body of evidence for an outcome. | P4,5 |
| **RESULTS** | | |  |
| Study selection | 16a | Describe the results of the search and selection process, from the number of records identified in the search to the number of studies included in the review, ideally using a flow diagram. | P5, 7 |
|  | 16b | Cite studies that might appear to meet the inclusion criteria, but which were excluded, and explain why they were excluded. | P5, 7 |
| Study characteristics | 17 | Cite each included study and present its characteristics. | P5, 7 |
| Risk of bias in studies | 18 | Present assessments of risk of bias for each included study. | P5, 7 |
| Results of individual studies | 19 | For all outcomes, present, for each study: (a) summary statistics for each group (where appropriate) and (b) an effect estimate and its precision (e.g. confidence/credible interval), ideally using structured tables or plots. | P5, 7 |
| Results of syntheses | 20a | For each synthesis, briefly summarise the characteristics and risk of bias among contributing studies. | P5, 7 |
|  | 20b | Present results of all statistical syntheses conducted. If meta-analysis was done, present for each the summary estimate and its precision (e.g. confidence/credible interval) and measures of statistical heterogeneity. If comparing groups, describe the direction of the effect. | P5, 7 |
|  | 20c | Present results of all investigations of possible causes of heterogeneity among study results. | P5, 7 |
|  | 20d | Present results of all sensitivity analyses conducted to assess the robustness of the synthesized results. | P5, 7 |
| Reporting biases | 21 | Present assessments of risk of bias due to missing results (arising from reporting biases) for each synthesis assessed. | P5, 7 |
| Certainty of evidence | 22 | Present assessments of certainty (or confidence) in the body of evidence for each outcome assessed. | P5, 7 |
| **DISCUSSION** | | |  |
| Discussion | 23a | Provide a general interpretation of the results in the context of other evidence. | P7, 9 |
|  | 23b | Discuss any limitations of the evidence included in the review. | P7, 9 |
|  | 23c | Discuss any limitations of the review processes used. | P7, 9 |
|  | 23d | Discuss implications of the results for practice, policy, and future research. | P7, 9 |
| **OTHER INFORMATION** | | |  |
| Registration and protocol | 24a | Provide registration information for the review, including register name and registration number, or state that the review was not registered. | P4 |
|  | 24b | Indicate where the review protocol can be accessed, or state that a protocol was not prepared. | P4 |
|  | 24c | Describe and explain any amendments to information provided at registration or in the protocol. | P4 |
| Support | 25 | Describe sources of financial or non-financial support for the review, and the role of the funders or sponsors in the review. | P9 |
| Competing interests | 26 | Declare any competing interests of review authors. | P9 |
| Availability of data, code and other materials | 27 | Report which of the following are publicly available and where they can be found: template data collection forms; data extracted from included studies; data used for all analyses; analytic code; any other materials used in the review. | P9 |

Table 1S: PRISMA checklist

| Outcome name | Number of laparoscopic studies included in the outcome | Number of robotic studies included in the outcome |
| --- | --- | --- |
| Operative time | 48 | 3 |
| Splenic flexure mobilization | 21 | 1 |
| Blood loss | 22 | 1 |
| Conversion to open surgery | 39 | 4 |
| Length of hospital stay | 52 | 4 |
| Ileus | 26 | 3 |
| Morbidity | 27 | 2 |
| Fever | 2 | 1 |
| Surgical site infection | 35 | 4 |
| Pneumonia | 25 | 2 |
| Sepsis | 6 | 1 |
| Anastomotic leak | 19 | 2 |
| Cardiac events | 48 | 2 |
| Reoperation | 21 | 1 |
| Direct comparison operative time | 2 | 2 |
| Direct comparison conversion to open approach | 3 | 3 |
| Direct comparison morbidity | 2 | 2 |
| Direct comparison anastomotic leak | 2 | 2 |

Table 2S: Number of studies contributing to each outcome

| No. | AMSTAR 2 Item | Rating (Yes / Partial /No) |
| --- | --- | --- |
| 1 | Did the research questions and inclusion criteria include the components of PICO? | Yes |
| 2 | Did the review have an explicit statement that the methods were established prior to the conduct of the review (e.g., a protocol)? | Yes |
| 3 | Did the review authors use a comprehensive literature search strategy? | Yes |
| 4 | Did the review authors perform study selection in duplicate? | Yes |
| 5 | Did the review authors perform data extraction in duplicate? | Yes |
| 6 | Did the review authors provide a list of excluded studies and justify the exclusions? | Yes |
| 7 | Did the review authors describe the included studies in adequate detail? | Yes |
| 8 | Did the review authors use a satisfactory technique for assessing the risk of bias (RoB) in individual studies? | Yes |
| 9 | Did the review authors report on the sources of funding for the studies included in the review? | Yes |
| 10 | If meta-analysis was performed, did the review authors use appropriate methods for statistical combination of results? | Yes |
| 11 | If meta-analysis was performed, did the review authors assess the potential impact of RoB in individual studies on the results? | Yes |
| 12 | Did the review authors account for RoB in individual studies when interpreting/discussing the results of the review? | Yes |
| 13 | Did the review authors provide a satisfactory explanation for, and discussion of, any heterogeneity observed in the results? | Yes |
| 14 | If they performed quantitative synthesis, did the review authors carry out an adequate investigation of publication bias? | Yes |
| 15 | Did the review authors report any potential sources of conflict of interest, including any funding they received for conducting the review? | Yes |
| 16 | Were the review authors’ conclusions supported by the results and did they consider the limitations of the included studies? | Yes |

Table 3S: AMSTAR 2 checklist
